# Impact of Computerized Physician Order Entry (CPOE) Coupled With Clinical Decision Support (CDS) on Radiologic Services

**DOI:** 10.7759/cureus.69470

**Published:** 2024-09-15

**Authors:** Mansour Almanaa

**Affiliations:** 1 Radiological Sciences Department, College of Applied Medical Sciences, King Saud University, Riyadh, SAU

**Keywords:** alert, alert fatigue, clinical decision support, computerized physician order entry, medical imaging, radiology

## Abstract

Medical imaging is an essential component of healthcare, enabling accurate diagnoses and facilitating effective treatment plans. However, the field is not without its challenges, including medical imaging errors, overutilization of procedures, and adverse reactions to contrast agents. This review explores the impact of computerized physician order entry (CPOE) systems coupled with clinical decision support (CDS) on radiologic services. By analyzing the findings from various studies, this paper highlights how CPOE coupled with CDS can significantly reduce inappropriate imaging, enhance adherence to clinical guidelines, and improve overall patient safety. The implementation of CPOE with CDS optimizes the utilization of radiologic procedures, thereby reducing healthcare costs and minimizing patients' exposure to unnecessary radiation. Despite its benefits, the adoption of CPOE with CDS encounters challenges such as high implementation costs, changes in workflow, and alert fatigue among healthcare providers. Addressing these challenges requires careful system design, including the customization of alerts to reduce override rates and improve the specificity of CDS recommendations. This review underscores the potential of CPOE with CDS to transform radiologic services, enhancing both the quality and safety of patient care. Further research is needed to explore the system's effectiveness in preventing adverse reactions to contrast media and to identify best practices for overcoming the barriers to its broader adoption.

## Introduction and background

Medical imaging exams are an important part of healthcare that have led to enhancements in the diagnosis and treatment of many health conditions. Medical imaging enables the visualization of internal organs, allowing physicians to examine, diagnose, and treat various medical conditions [1]. The field has evolved rapidly with the advancement of technology and computing [1]. Following the ALARA principle ("As Low As Reasonably Achievable") is crucial for healthcare providers, emphasizing the delivery of high-quality care while minimizing patients' exposure to unnecessary radiation [2]. However, medical imaging is not an exception to other healthcare services. Radiologic medical imaging suffers from several issues that can affect the quality of care, patient safety, and healthcare expenses.

Several issues could occur in medical imaging services. Medical imaging errors are one of the issues in radiologic services and are not uncommon [3]. The types of errors that could happen in radiologic medical imaging include wrong-procedure errors, wrong-patient errors, and wrong-site errors [3]. Unnecessary or inappropriate radiologic procedures are also a major problem in medical imaging services. They increase costs to the government and patients and expose patients to unnecessary doses of radiation [4,5]. Materials that are administered to patients to enhance images could be problematic as well. They can cause adverse reactions and, in rare cases, death [6].

A computerized physician order entry (CPOE) system coupled with clinical decision support (CDS) could be an effective way to reduce the issues and improve healthcare services in radiologic medical imaging [7-20]. This paper aims to synthesize existing evidence on the effectiveness of integrating CDS within a CPOE system to enhance the quality of radiologic services.

## Review

Methods

This review was conducted and reported in accordance with the PRISMA Extension for Scoping Reviews (PRISMA-ScR) guidelines [21]. Articles for this review were sourced from Google Scholar, PubMed, and Ovid. The search keywords included "CPOE," "computerized physician order entry," "CDS," "clinical decision support," "radiology," "radiologic imaging," "medical imaging," and "imaging." Boolean operators "OR" and "AND" were utilized to combine these keywords, ensuring comprehensive coverage of relevant studies. Additionally, the references of the included articles were reviewed to identify other relevant studies.

**Figure 1:**
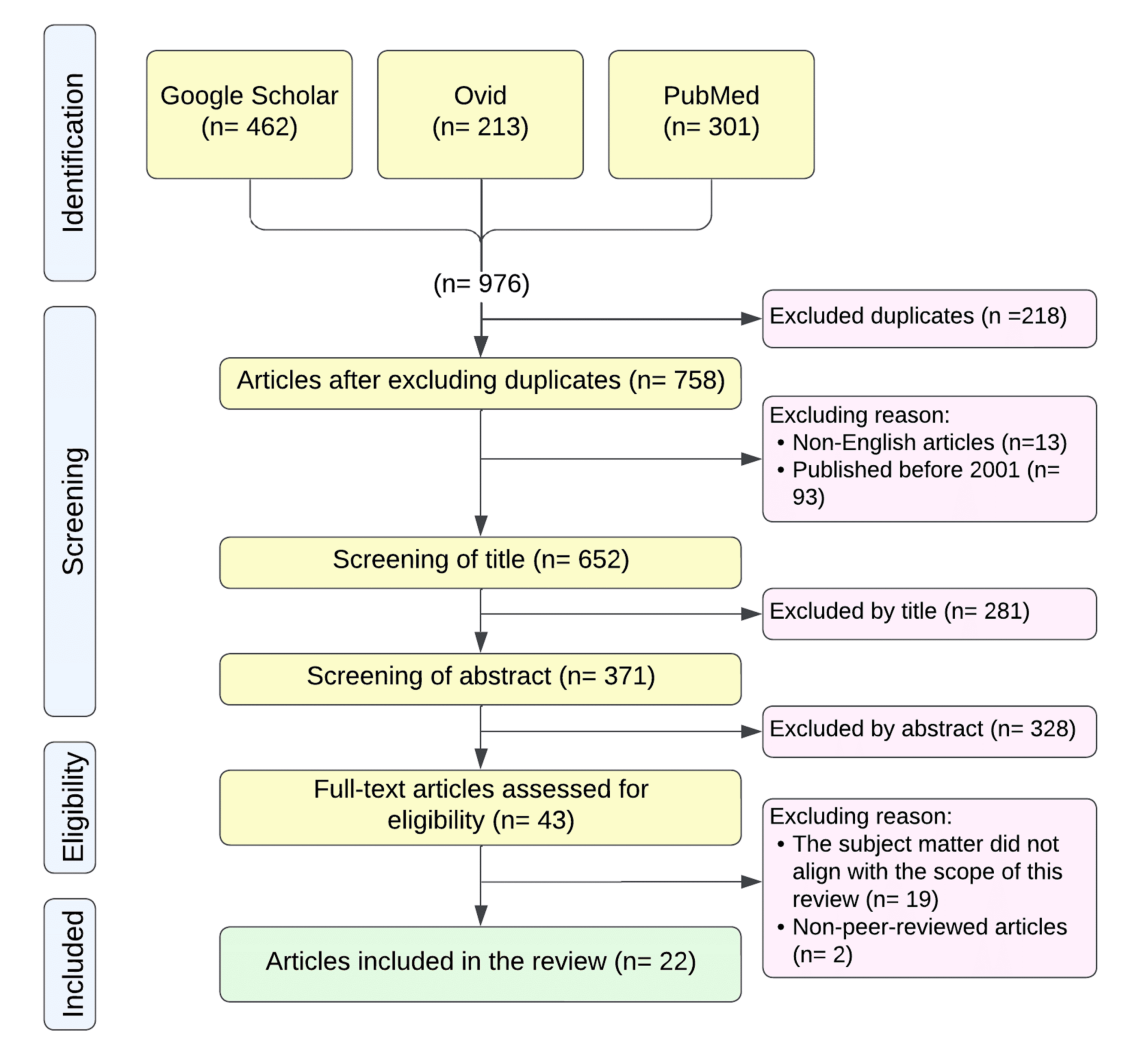
Flowchart of the article selection process for the review

The inclusion criteria required that articles be peer-reviewed, in English, published in or after 2001, and relevant to the scope of this paper, the impact of CPOE with CDS on radiologic services. Restricting the review to English-language articles ensures accessibility and comprehension, given that English is the predominant language of scientific publications, thus maintaining the quality and reliability of the review by focusing on peer-reviewed and reputable sources. The chosen timeframe of 2001 to 2024 captures the significant advancements in CPOE and CDS systems over the past two decades, reflecting contemporary practices and the most recent evidence. This criterion also aids in maintaining the review's scope and manageability, allowing for a focused and rigorous analysis without being overwhelmed by an excessive volume of literature. The search and selection process is detailed in Figure 1, illustrating the steps from the initial search to the final inclusion of articles.

Results

The search yielded 976 articles: 462 from Google Scholar, 213 from Ovid, and 301 from PubMed. Duplicate and non-English articles were excluded, as well as those published before 2001, resulting in 652 articles. Based on the titles, 281 articles were excluded, leaving 371 articles. After reviewing the abstracts, 328 articles were excluded, leaving 43 articles. Following a full review, 21 articles were excluded because their primary subject matter was not aligned with the scope of this review or because they were non-peer-reviewed, leaving a total of 22 articles included in the review. These 22 articles served as the primary sources for this review, while the remaining articles were used to support the discussion. A summary of the included articles is provided in Table 1.

**Table 1:** Summary table of reviewed articles CDS: clinical decision support, CPOE: computerized physician order entry, US: United States, CT: computed tomography, CTPA: computed tomography pulmonary angiography, MRI: magnetic resonance imaging, ED: emergency department, ESR: European Society of Radiology

No.	Article title	Authors	Year of publication	Objective/purpose	Methodology	Key findings	Relevance to CPOE and CDS in radiologic services
1	The effects on clinician ordering patterns of a computerized decision support system for neuroradiology imaging studies [7]	Sanders DL, Miller RA	2001	To evaluate the impact of a CDS system on clinician ordering patterns for neuroradiology imaging.	Retrospective pre-post analysis.	Increased appropriateness of neuroradiology imaging orders.	Demonstrates the effectiveness of CDS in specific radiologic subspecialties within CPOE.
2	Assessment of radiological referral practice and effect of computer-based guidelines on Radiological requests in two emergency departments [8]	Carton M, Auvert B, Guerini H, et al.	2002	To assess the impact of computer-based guidelines on radiology referral practices in emergency departments.	Comparative study across two hospitals.	Improved adherence to guidelines and reduced inappropriate referrals.	Highlights the role of CDS in enhancing radiologic referrals through CPOE systems.
3	A utilization management intervention to reduce unnecessary testing in the coronary care unit [9]	Wang TJ, Mort EA, Nordberg P, et al.	2002	To evaluate the effectiveness of utilization management interventions in reducing unnecessary testing.	Intervention study with before-and-after comparison.	Reduced unnecessary testing in coronary care units.	Relevant to understanding the broader impact of CPOE with CDS on various medical specialties.
4	Effect of computerized order entry with integrated decision support on the growth of outpatient procedure volumes: seven-year time series analysis [10]	Sistrom CL, Dang PA, Weilburg JB, et al.	2009	To analyze the long-term impact of CPOE with integrated CDS on outpatient procedure volumes.	Time series analysis over seven years.	Gradual increase in appropriate outpatient imaging procedures.	Demonstrates the long-term benefits of CPOE with CDS on procedural growth and appropriateness.
5	Meaningful use of computerized prescriber order entry [11]	Classen D, Bates DW, Denham CR	2010	To discuss the meaningful use criteria for CPOE and its implications for healthcare delivery.	Review and policy analysis.	CPOE meaningful use can improve patient safety and reduce errors.	Relevant to understanding the regulatory and policy framework for CPOE with CDS.
6	Increasing the appropriateness of outpatient imaging: effects of a barrier to ordering low-yield examinations [12]	Vartanians VM, Sistrom CL, Weilburg JB, et al.	2010	To assess the impact of barriers to ordering low-yield imaging exams on outpatient imaging practices.	Analysis of imaging order patterns before and after barrier implementation.	Significant reduction in low-yield exams ordered.	Highlights how CDS can guide better decision-making in outpatient imaging through CPOE systems.
7	Electronic decision support for diagnostic imaging in a primary care setting [13]	Curry L, Reed MH	2011	To examine the impact of electronic decision support on diagnostic imaging in primary care.	Comparative study of imaging orders before and after CDS implementation.	Increased appropriateness of imaging orders in primary care.	Demonstrates the potential of CDS in improving imaging decisions.
8	Standard practices for computerized clinical decision support in community hospitals: a national survey [14]	Ash JS, McCormack JL, Sittig DF, et al.	2012	To survey the adoption and standard practices of CDS in community hospitals across the US.	National survey of hospital practices.	Wide variability in CDS practices; need for standardization identified.	Provides context on the varying implementation of CPOE and CDS in different hospital settings.
9	Clinical decision support systems for utilization of CT in the emergency department [15]	Ip IK, Drescher FS	2012	To evaluate the role of CDS in managing the use of CT scans in the emergency department.	Review of clinical studies and systems analysis.	CDS can reduce unnecessary CT scans and improve adherence to guidelines.	Relevant to understanding the application of CDS in radiologic services within CPOE systems.
10	Effect of computerized clinical decision support on the use and yield of CT pulmonary angiography in the emergency department [16]	Raja AS, Ip IK, Prevedello LM, et al.	2012	To assess the impact of CDS on the utilization and diagnostic yield of CTPA in the ED.	Retrospective analysis comparing pre- and post-CDS implementation data.	Reduced the number of unnecessary CTPA exams without affecting diagnostic yield.	Highlights how CPOE with CDS can optimize the use of high-radiation imaging studies.
11	The effect of computerized provider order entry systems on clinical care and work processes in emergency departments: a systematic review of the quantitative literature [17]	Georgiou A, Prgomet M, Paoloni R, et al.	2013	To systematically review the impact of CPOE systems on clinical care and workflows in emergency departments.	Systematic review of quantitative studies.	Mixed effects on workflow but generally improved order accuracy and patient safety.	Provides insights into how CPOE with CDS can be optimized to balance workflow efficiency and safety.
12	Requiring clinical justification to override repeat imaging decision support: impact on CT use [18]	O'Connor SD, Sodickson AD, Ip IK, et al.	2014	To examine the effects of requiring clinical justification for overriding CDS alerts on repeat CT imaging orders.	Prospective study with data collection before and after intervention.	Decreased the number of unnecessary repeat CT scans.	Demonstrates the importance of structured CDS in preventing redundant imaging in CPOE systems.
13	Radiology reporting: a closed-loop cycle from order entry to results communication [19]	Weiss DL, Kim W, Branstetter BF, Prevedello LM	2014	To assess the complete cycle of radiology reporting from order entry to results communication.	Review of existing systems and workflows in radiology departments.	Identified gaps in communication and reporting; proposed a closed-loop system to ensure accuracy.	Relevant in ensuring that CPOE and CDS systems contribute to a comprehensive radiology workflow.
14	Impact of clinical decision support on head computed tomography use in patients with mild traumatic brain injury in the ED [20]	Ip IK, Raja AS, Gupta A, Andruchow J, Sodickson A, Khorasani R	2015	To evaluate the impact of CDS on the utilization of head CT for patients with mild traumatic brain injury in the emergency department.	Retrospective study analyzing CT scan usage before and after CDS implementation.	Significant reduction in unnecessary head CT scans.	Demonstrates how CDS within CPOE can reduce unnecessary imaging and improve decision-making.
15	Radiology order decision support: examination-indication appropriateness assessed using 2 electronic systems [22]	Schneider E, Zelenka S, Grooff P, et al.	2015	To compare two CDS systems in evaluating the appropriateness of MRI and CT orders.	Retrospective analysis of 2,000 MRI and CT orders across two CDS systems.	The study found notable differences in appropriateness ratings between the systems, influenced by their design and implementation.	Highlights the need for careful selection and configuration of CDS in CPOE systems to ensure accurate imaging appropriateness assessments.
16	Provider feedback about imaging appropriateness using scores from order entry decision support: raw rates misclassify outliers [23]	Sistrom CL, Weilburg JB, Dreyer KJ, Ferris TG	2015	To evaluate how feedback on imaging appropriateness based on order entry decision support scores affects provider behavior.	Retrospective analysis of outpatient imaging orders over five years, using statistical methods to identify factors influencing low-utility imaging rates.	Significant variation in low-utility imaging rates among providers, with caution advised in using raw rates for feedback.	Emphasizes the need for accurate metrics in CDS within CPOE systems to avoid misclassification and improve imaging practices.
17	Integrity of clinical information in computerized order requisitions for diagnostic imaging [24]	Lacson R, Laroya R, Wang A, et al.	2018	To evaluate the accuracy and integrity of clinical information in CPOE systems for diagnostic imaging.	Data quality analysis of CPOE entries.	Identified issues in data integrity affecting CDS effectiveness.	Highlights the importance of accurate data entry in CPOE systems for effective CDS functioning.
18	Can emergency department provider notes help to achieve more dynamic clinical decision support? [25]	Rousseau JF, Ip IK, Raja AS, Schuur JD, Khorasani R	2020	To investigate whether integrating provider notes in the ED can enhance CDS functionality.	Prospective observational study.	Provider notes enhanced the specificity of CDS alerts.	Relevant to improving the dynamic nature of CDS in radiologic services within CPOE systems.
19	A systematic analysis of the optimization of computerized physician order entry and clinical decision support systems: a qualitative study in English hospitals [26]	Wiegel V, King A, Mozaffar H, et al.	2020	To analyze the optimization of CPOE and CDS systems in English hospitals.	Qualitative study using interviews and observations.	Identified best practices and challenges in CPOE and CDS implementation.	Provides insights into optimizing CPOE with CDS in healthcare settings.
20	Evaluation criteria for the effects of decision support integrated into computerized provider order entry system: a scoping review [27]	Karajizadeh M, Zand F, Vazin A, et al.	2022	To review and identify evaluation criteria for CDS integrated into CPOE systems.	Scoping review of evaluation studies.	Proposed a set of criteria for evaluating CPOE with CDS.	Relevant to understanding how to assess and improve CPOE with CDS in radiologic services.
21	Analytics to monitor local impact of the protecting access to Medicare Act’s imaging clinical decision support requirements [28]	Valtchinov VI, Murphy SN, Lacson R, et al.	2022	To monitor the impact of the Protecting Access to Medicare Act’s imaging CDS requirements.	Analytics-based study using hospital data.	Provided insights into compliance and effectiveness of CDS requirements.	Relevant to understanding the regulatory impacts on CDS and CPOE in radiologic services.
22	Medical imaging decision and support (MIDAS): study protocol for a multi-centre cluster randomized trial evaluating the ESR iGuide [29]	Dijk SW, Kroencke T, Wollny C, et al.	2023	To outline the protocol for a trial evaluating the ESR iGuide for medical imaging decision support.	Protocol for a cluster randomized trial.	Anticipated to reduce inappropriate imaging and improve guideline adherence.	Relevant to the development and evaluation of new CDS tools within CPOE systems.

Synthesis of the studies

Issues in Radiologic Services

Radiologic services are essential to modern healthcare, but they encounter several significant challenges that can impact both patient safety and the overall quality of care [1,3]. Foremost among these is the issue of medical imaging errors. These errors can occur in various forms, including wrong-procedure errors, where the incorrect imaging procedure is performed; wrong-patient errors, where the wrong patient's imaging is conducted; and wrong-site errors, where imaging is performed on the incorrect part of the body [3]. Such errors may lead to misdiagnoses, delayed treatment, and even unnecessary surgical interventions. According to the Pennsylvania Patient Safety Authority, several factors contribute to errors in radiologic services. One significant cause is the inaccurate entry of radiologic orders, such as when a healthcare provider fails to specify whether the exam should be conducted with contrast media [3,24]. This type of error accounts for half of the errors in radiologic services [3]. Another common cause, responsible for approximately one-third of the errors, is the failure to confirm the patient's identity, which can result in exposing the wrong patient to radiation [3]. Additionally, errors can occur due to the failure to verify the site or procedure of the radiologic exam, a factor that contributes to one-fifth of the errors in radiologic services [3].

Another critical issue in radiologic services is the overutilization of imaging procedures [7,12]. Overutilization involves performing unnecessary or inappropriate imaging exams that do not contribute to the patient's care but instead increase healthcare costs and expose patients to unnecessary radiation [4,5]. The use of unnecessary imaging procedures not only wastes resources but also puts patients at risk for adverse outcomes, including radiation-induced injuries and adverse reactions to contrast agents used during imaging [4,6].

Results from a study conducted by Dehn et al. suggest that 30-40% of medical imaging exams are inappropriate [30]. Exposure to high doses of radiation may increase the risk of developing cancer [31,32]. In the United States, the number of CT scans performed increased from 3 million in 1980 to 67 million in 2007, and it is increasing by 10% every year [4]. Approximately 29,000 cancers every year could be caused by CT scans [33]. It is estimated that two to three head CT scans done on children aged younger than 15 years old can triple the risk of brain tumors [34]. The availability and convenience of ordering diagnostic procedures such as medical imaging exams may increase the risk of overutilization [35]. Therefore, preventing unnecessary exposure to radiation is a critical priority.

In addition to errors and overutilization, there is the issue of adverse reactions to contrast media. These agents are commonly used to enhance the visibility of specific structures or fluids within the body during imaging [36], but they can cause allergic reactions, ranging from mild to severe, and in rare cases, they can be life-threatening [6,37]. Managing these reactions requires prompt recognition and intervention, and the risk of such reactions must be carefully weighed against the potential benefits of the imaging procedure. Despite the existence of protocols for the safe administration of contrast media, incidents of adverse reactions still occur, highlighting the need for enhanced safety measures and improved risk assessment tools [6]. Incorporating CDS into a CPOE system presents a promising approach to enhancing the quality of medical imaging services. However, this area requires further research.

What Is a CPOE System With CDS?

The CPOE component is an electronic system that replaces traditional methods of ordering medications, laboratory tests, imaging studies, and other diagnostic procedures. Instead of handwriting or verbally communicating orders, healthcare providers enter orders directly into an electronic health record (EHR) system [15,38]. This not only reduces the risk of errors associated with handwriting and miscommunication but also ensures that orders are transmitted directly to the appropriate department (e.g., pharmacy, laboratory, radiology) for execution [38]. To maximize the benefits of utilizing CPOE, it should be combined with the CDS system. CDS is designed to assist healthcare providers in making informed decisions about patient care [39].

The CDS component complements the CPOE system by providing real-time, evidence-based guidance to healthcare providers as they enter orders [15]. CDS tools can include alerts, reminders, clinical guidelines, diagnostic support, and drug interaction checks, all aimed at improving the safety and quality of patient care [10]. For instance, when a physician orders a CT scan for a patient with mild symptoms, the CDS system may generate an alert indicating that, based on the patient's condition and current clinical guidelines, the CT scan may not be necessary. This type of guidance enables healthcare providers to make more informed decisions, reduce unnecessary procedures, and adhere to best practices [10,13].

Patient-specific recommendations or alerts are generated by software algorithms when the characteristics of a patient match a computerized knowledge base. The characteristics of a patient can be entered manually or retrieved from an EHR [39]. These computer-generated recommendations or alerts are displayed on the healthcare provider’s screen when there appears to be an issue. The accuracy of clinical information within CPOE is crucial for the effective operation of CDS systems, which in turn helps to minimize diagnostic errors and enhance patient safety [24].

CPOE With CDS as a Means to Eliminate Issues and Improve Quality in Radiologic Services

Medical imaging procedures are evolving rapidly, becoming increasingly complex over time [19]. As a result, many healthcare providers find it difficult to select the most appropriate radiologic exam for their patients, leading to the selection of inappropriate or suboptimal imaging procedures [19]. When CPOE is coupled with CDS and applied to radiologic services, it can guide healthcare providers toward more optimized utilization of medical imaging examinations [9,15,22,23,25,27,29].

Currently, numerous clinical decision rules related to radiologic services, such as the Ottawa ankle and knee rules [40,41], have been developed to assist healthcare providers in their decision-making. However, applying these rules in clinical practices remains challenging [42]. CDS within a CPOE system can facilitate the application of these rules and help healthcare providers make more appropriate decisions [15,27]. Evidence suggests that CPOE with CDS improves healthcare providers' adherence to guidelines and rules [8,11,27]. When these guidelines are followed, the number of inappropriate and unnecessary medical imaging examinations significantly decreases [7,9,20,27,43,44], which subsequently lowers healthcare expenses and reduces radiation exposure to patients [8,20,45]. For instance, adherence to head CT scan guidelines alone could reduce healthcare expenditures in the United States by $120 million annually [45]. Additionally, a CPOE system with CDS not only reduces unnecessary medical imaging procedures but also enhances healthcare quality without increasing the risk of delayed diagnoses [20]. Furthermore, administrators can utilize the CPOE system's database to review historical physician orders, identify patterns of undesirable ordering, and provide targeted advice to improve decision-making [19].

Incorporating CDS within CPOE can significantly improve healthcare quality by reducing inappropriate medical imaging exams and decreasing the utilization of high-radiation radiologic procedures [27,28,46]. A study conducted by Bairstow et al. [46] revealed that CDS decreased inappropriate diagnostic imaging procedures by 13%; moreover, the finding of their study showed that 35% of the medical imaging procedures performed before the CDS implementation were not adherent to guidelines, whereas only 22% of which were not adherent to guidelines after the implementation of CDS [46]. Patients are exposed to very high radiation doses when they undergo CT scans [47]. CDS can help reduce the utilization of CT angiography, which means decreasing patients’ exposure to unnecessary high radiation doses [10,16,48].

Radiographic contrast media used in medical imaging are highly valuable, as they can significantly enhance the images of body organs produced by various radiologic imaging procedures. However, these contrast agents can also cause adverse reactions, including life-threatening ones [6,37]. It is crucial to identify patients at high risk for contrast media reactions, such as those with a previous reaction to contrast media, asthma, or food allergies [49] so that they can receive special care to prevent such reactions. CPOE with CDS could assist in identifying patients at high risk for reactions to contrast agents. Currently, there appears to be no existing study that specifically examines the impact of CPOE coupled with CDS on identifying patients at high risk for contrast media reactions. Several studies have explored the effectiveness of CPOE with CDS in reducing adverse drug reactions, with findings indicating that CPOE with CDS significantly reduced adverse drug events [17,50-52]. It is reasonable to suggest that CPOE with CDS could prevent contrast media reactions in the same way it has been shown to prevent adverse drug reactions. Further research is needed to explore this area and validate these potential benefits.

Problem of CDS Alerts Override

CDS alerts are specific types of CDS automation designed to notify healthcare providers about critical information. Alerts can be either interruptive or simply informative, appearing somewhere on the healthcare provider’s screen [53]. These alerts can be highly important and beneficial. In a study conducted by Persell et al. [54], the results showed that CDS alerts helped boost physician performance and enhanced patient care. However, it has been observed that many physicians tend to override these alerts, with override rates occurring in 49-98% of cases [55].

In CPOE systems, alert overriding is a common issue. There are two types of overriding: those that are justified due to valid reasons and those that are unjustified and, therefore, incorrect. Incorrect overriding can result in serious health issues for patients [22,25,29]. Such actions should, therefore, be avoided whenever possible. Most of the time, however, incorrect overriding results from alert fatigue, which arises from a continuous flow of alerts that are sometimes irrelevant [26-28,56]. As physicians receive more alerts, they become overwhelmed, leading to a decrease in responsiveness, a phenomenon known as alert fatigue, which is the major cause of alert override [57,58].

Alert fatigue can be defined as a decline in the responsiveness of healthcare providers as the number of simultaneous alerts increases [57-59]. Healthcare providers have limited ability to modify alerts to ensure only important ones are displayed. This limitation is due to concerns from vendors and designers about potential liability if they allow healthcare providers to remove or modify an alert that could have prevented a medical error, thereby restricting the ability to adjust or modify alerts [26,58].

Alert Fatigue and CDS Alerts Within CPOE

In some cases, incorrect overriding can result in serious consequences. Therefore, many CDS systems are designed with a high sensitivity rate, which often comes at the expense of specificity [60]. Systems with lower specificity are more likely to be overridden as physicians receive more false-positive alerts and become desensitized to the importance of these alerts [14,61]. False-positive alerts increase the cognitive load and consume more time as physicians try to assess the relevance of the alerts [60].

Although the issue of alert fatigue is well documented, the solution is not straightforward. The high number of overridden alerts suggests that many alerts should not be triggered; however, studies have shown that physicians may disagree on which alerts can be safely removed [62,63]. Overridden alerts are not always irrelevant. Some of the reasons for overriding alerts include "alert well known," "alert not serious," "alert not needing (additional) action," or "the effects of the combination were monitored or intended" [63].

Another attempt to improve the quality of triggered alerts was made through machine learning. Lee et al. [62] applied machine learning to predict which alerts could be safely removed based on previously overridden alerts. The method predicted the overridden alerts with 91-96% accuracy. The study also emphasized the importance of patient safety when filtering alerts, as some of the filtered alerts might be important. Additionally, the study showed that reducing the number of alerts triggered may compromise quality [62]. To reduce override rates of CDS alerts within CPOE, the alerts need to be carefully constructed, considering both sensitivity and specificity [62].

Recommendations to Design Good CDS Alerts Within CPOE

Alert override is a significant problem because, as physicians override irrelevant alerts, they may also end up overriding important alerts due to a lack of responsiveness [26,64]. CDS alerts within a CPOE system should be designed in a way that encourages healthcare providers to follow the information presented in them [22,23,26,29].

Several factors should be considered to ensure that CDS alerts are effective. First, CDS alerts should be reliable and accurate so that healthcare providers can trust them [65]. Approximately 41% of physicians do not trust the information presented in CDS alerts [14]. Second, in interviews conducted by Birmingham et al. [66], healthcare providers expressed a desire for CDS alerts to include the patient’s medical history so that physicians do not have to search for the required health information. Moreover, CDS alerts should remain on the screen without interrupting the workflow until the physician is ready to act on them. CDS alerts should not require multiple responses or immediate actions, and they should not interrupt or disturb the physician’s workflow; however, they should be noticeable [67]. Third, for potentially serious clinical circumstances, the CDS alert should use a “hard stop” to prevent healthcare providers from proceeding with orders that conflict with the evidence presented in the alert [50,56,60,68]. Fourth, the CDS alert should be placed in an area that is within the users’ field of operation. The viewing angle should be at most 50 degrees down from the straight line of normal view, allowing targets to be observed without eye movement [60]. Fifth, CDS alerts should be tiered based on the level of clinical severity. This can be achieved by using colors to represent different levels of clinical severity. For instance, red and orange could represent high levels of danger, while green and blue could represent lower levels [69,70]. Additionally, alerts should be designed to be as distinct as possible, making them easy to differentiate [67]. Sixth, CDS alerts should be actionable, meaning that healthcare providers can take action directly through the alert. Passive alerts are not as effective as actionable alerts [13,71]. Seventh, CDS alerts that are not specific to the clinical context are less effective. Therefore, alerts should be tailored to the patient under examination [15,72-74]. Finally, if a healthcare provider wishes to override a serious CDS alert, they should justify or obtain authorization. CDS alerts should either include a space for the provider to enter a written justification [18,75] or require authorization from a responsible clinician to override them [12].

Barriers to Implement CPOE With CDS

Despite the benefits of the CPOE system with CDS, its adoption has been slow [76]. There are a number of barriers to CPOE adoption, summarized in Table 2. The primary barrier to CPOE adoption is the cost [15,77-79]. In a study published in 2005, it was estimated that the implementation of a CPOE system could cost between $1.3 million and $4.4 million [80]. Another financial barrier is that reducing the number of medical imaging examinations could lead to lower profits for private-for-profit healthcare centers, whose revenues primarily depend on fees for services [15]. Other barriers include the time required to use the system [15], the changes in workflow [78,81], the need for training for healthcare professionals [79,82], and the complexity of the system [11,79,83].

**Table 2:** Summary of CPOE adoption barriers CPOE: computerized physician order entry

Barrier	Detail
Cost	The start-up cost of a CPOE system could be a significant obstacle
Time	CPOE may require physicians to spend substantial time away from their patients
Changes in workflow	Changes in workflow and increased workload can be a barrier to CPOE adoption
Training	Training may be required for healthcare providers to effectively use the CPOE system
Complexity	The complexity of the CPOE system could be a barrier to its implementation

Practical Recommendations for Implementing CPOE With CDS in Radiologic Services

Based on the findings of this review, several practical recommendations can be made for healthcare organizations considering the implementation of CPOE with CDS in radiologic services. First, institutions should prioritize user training and ongoing support to reduce the learning curve and enhance adoption rates. Second, customizing CDS alerts to minimize alert fatigue and increase relevance can significantly improve user engagement and effectiveness. Lastly, organizations should consider the initial and ongoing costs of implementing these systems and explore funding options or phased implementations to manage financial constraints. These steps can help maximize the benefits of CPOE with CDS while mitigating the challenges identified in this review.

Limitations of This Review

While this review provides a comprehensive analysis of the impact of CPOE with CDS on radiologic services, several limitations must be acknowledged. First, the review was limited to studies published in English, which may exclude relevant research in other languages. Second, the variation in study designs and populations among the included studies makes it challenging to generalize the findings across all healthcare settings. Lastly, the rapidly evolving nature of CPOE and CDS technologies means that some of the findings may not fully capture the latest advancements in this field. Future research should aim to address these limitations by including a broader range of studies and considering the latest technological developments.

## Conclusions

Even with all the advancements in radiologic medical imaging, certain issues still persist. Medical imaging errors, unnecessary exposure to radiation, and adverse reactions to contrast agents are some of the problems that can occur in radiologic services. The studies included in this review suggest that CPOE with CDS can have a significant impact on the services provided in radiologic medical imaging. Through a CPOE system with CDS, healthcare providers could deliver optimal care, reduce issues in medical imaging, and lower healthcare expenses.

On the other hand, CPOE with CDS also has its challenges, such as the overriding of CDS alerts by healthcare providers, which could result in serious consequences. These problems can be eliminated by designing effective CDS alerts that are reliable, accurate, and actionable. Barriers to the adoption of the CPOE system with CDS for radiologic services need to be thoroughly studied to ensure successful implementation of the system.

This review highlights the transformative potential of CPOE with CDS in enhancing the quality and safety of radiologic services. However, several areas require further investigation to fully realize this potential. Future research should focus on evaluating the system's effectiveness in real-world clinical settings, where variables such as workflow integration, user adoption, and patient outcomes can be more accurately assessed. Additionally, more studies are needed to develop and test strategies to mitigate the challenges identified in this review, including high implementation costs, alert fatigue, and the need for user-friendly interface designs. Research should also explore the long-term impact of CPOE with CDS on patient safety, particularly in preventing adverse reactions to contrast media. Addressing these gaps will be crucial for optimizing the use of CPOE with CDS in radiologic services and ensuring its broader adoption in healthcare systems.
